# What’s the catch? Urine sample collection from young pre-continent children: a qualitative study in primary care.

**DOI:** 10.3399/bjgpopen20X101060

**Published:** 2020-08-05

**Authors:** Jonathan Kaufman, Lena Sanci, Meredith Temple-Smith

**Affiliations:** 1 General Practice, Faculty of Medicine Dentistry and Health Sciences, University of Melbourne, Melbourne, Victoria, Australia; 2 General Paediatrics, Sunshine Hospital, St Albans, Australia; 3 Health Services Research Group, Murdoch Childrens Research Institute, Parkville, Victoria, Australia

**Keywords:** urine specimen collection, infant, urinary tract infections, paediatrics, general practice, primary health care

## Abstract

**Background:**

Urinary tract infections (UTIs) are common in young pre-continent children, but collecting urine samples is challenging. Collection methods all have limitations and international guidelines have conflicting recommendations. Choice of method must balance time, resources, invasiveness, reliability, and contamination. Evidence from primary care clinicians is limited regarding barriers and enablers to sample collection, and what factors inform the choice and use of different sample collection methods.

**Aim:**

To understand the barriers and enablers to collecting urine samples from young pre-continent children in primary care.

**Design & setting:**

An exploratory qualitative study performed in primary care in Australia.

**Method:**

Semi-structured interviews explored the topic of collecting a urine sample from a child aged 6 months who presented with a fever. The interviews were undertaken with 21 GPs and four practice nurses (PNs) until data saturation was reached. Interviews were audiorecorded, transcribed verbatim, coded, and underwent content and thematic analysis.

**Results:**

Five main themes emerged including: the clinician’s knowledge and expertise; patient characteristics; parent or carer’s understanding and motivation; the collection process itself; and likely outcome of the chosen method. Non-invasive methods were strongly favoured; although, clean catch was considered time-consuming and urine bags were known to be often contaminated. Invasive methods (for example, catheterisation or suprapubic aspiration [SPA]) were rarely performed outside of remote settings. Key barriers included time and space constraints in clinics, and key enablers included parental motivation, education handouts, and voiding stimulation methods.

**Conclusion:**

This study has identified key barriers and enablers to inform education, policy, and future research for urine sample collection from pre-continent children in primary care. Guideline recommendations must consider the primary care context to ensure they are relevant and suited to real-world practice.

## How this fits in

Collecting a urine sample to diagnose or exclude UTI in a young child can be challenging, and existing collection methods all have limitations. This qualitative study found that non-invasive collection methods were strongly preferred in primary care, although clinicians thought that clean catch could be time-consuming and urine bag samples were often contaminated. Barriers to collection included the time and resource constraints of the primary care setting, and enablers included parental motivation to collect samples and voiding stimulation methods. The ideal method would be fast, gentle, and simple, with low contamination. Further research is needed to optimise the speed and success, and reduce contamination, of non-invasive collection.

## Introduction

UTIs are common in early childhood, affecting 2%–6% of febrile young children in UK primary care.^1,2^ If untreated, UTI can cause significant complications, including sepsis, meningitis, and renal scarring. However, clinical signs are often non-specific, and overlap with many viral infections as well as other bacterial infections such as otitis media. As a result, UTI may not be considered or evaluated on initial presentation, and so the diagnosis is often delayed or missed in primary care.^3,4^ Therefore, UK guidelines recommend that any young child with signs and symptoms that could suggest UTI, or unexplained fever, should have a urine sample tested.^5^

Collecting a urine sample from a young pre-continent child is challenging, and limitations exist with all current urine collection methods. These include non-invasive (for example, urine bags, pads, or clean catch) and invasive (for example, catheter or SPA) methods. Once the decision to collect a sample has been made, choosing the method of urine sampling involves balancing the pros and cons related to time, resources, experience, invasiveness, reliability, and contamination rates.^6^

Australian and UK guidelines recommend clean catch as the first-line collection method,^5,7^ but as few as 20% of GPs use this method.^8^ Several guidelines specifically discourage the use of urine bags for culture owing to high contamination and false positive rates,^6,7,9^ but they are often favoured in primary care.^8,10^ Over 30% of children aged <2 years are diagnosed and treated for UTI without subsequent culture confirmation in some settings,^11–16^ despite recommendations this should occur when UTI is suspected.^5–7,9^

What is not well characterised is the reason for these choices, or what challenges are faced in collecting urine samples from young children in primary care. Limited evidence regarding barriers to collection comes mostly from studies in the hospital and emergency department (ED) setting,^10,17^ or surveys of parents.^18,19^ While these data suggest that practical difficulties are common, primary care has unique time and resource constraints compared with the hospital setting. Two questionnaire-based studies in primary care from 1997 partly identified some of these barriers, such as practical problems with collection,^14,15^ but did not explore different collection methods in detail.

To the authors' knowledge, there are no previous studies using interview-based qualitative research regarding a clinician’s choice and use of urine sample collection methods for young children, and the practicalities of collection, in the primary care setting. There is a need for further primary care research to ensure guideline recommendations are relevant to real-world practice. Qualitative research is particularly suited for the context where little is known about a topic, as it allows issues to be examined in depth.^20^ Therefore, the aim of this study was to explore the barriers and enablers to collecting urine samples from young pre-continent children in the primary care setting.

## Method

### Setting and recruitment

In the Australian primary healthcare system most practices are privately owned by groups of GPs or corporations. The national government health system, Medicare, funds or subsidises most consultations and services, but around 30% of Australians have out-of-pocket costs from seeing a GP.^21^

Purposive sampling was used to recruit a representative range of participants to broadly reflect the spectrum of the Australian primary care workforce by age, sex, rurality, and type of clinic. Australian GPs and PNs were recruited via the Victorian primary care practice-based Research and Education Network (VicReN),^22^ and professional networks. VicReN is a research and education network enabling collaboration between over 600 GP practices and the University of Melbourne Department of General Practice. Thirty-five practices and individual GPs were contacted by email or phone and invited to participate.

### Interviews

Participants gave written consent to participate. Semi-structured interviews were conducted face-to-face or by telephone between November 2018 and April 2019 until data saturation was reached. An interview guide included demographic questions followed by a scenario-based question related to a hypothetical clinical case, with further open questions exploring the topics outlined in Box 1. The scenario situated the conversation in the context of sample collection, once the decision to collect a sample had been made. The lead researcher conducting the interviews was a paediatrician and PhD candidate with research expertise regarding paediatric UTI, urine sample collection, and voiding stimulation methods.^23–25^

Box 1 **Interview guide**

**Scenario**

You see a febrile, 6-month-old child with fever and vomiting. On examination there is no focus for their fever, and they are not otherwise unwell.

The child is still feeding normally. The parents report the urine has a different smell than usual.

Would you collect a urine sample as part of their care?

**Topics**

Preferred method of collecting urine samples from young children

Why that method was preferred

Factors that facilitate sample collection (enablers)

Factors that obstruct sample collection (barriers)

Other collection methods considered and reasons for preferences

No financial incentives were offered to individual participants, but participating clinics were offered a 30-minute child health education session provided by the lead researcher.

Interviews lasted 14–36 minutes (mean 24 minutes) and were audiorecorded, transcribed verbatim, de-identified, and checked. Reflexive field notes were made following each interview.

### Analysis

NVivo (version 12) was used to code transcripts and organise data. Data were analysed using an inductive-deductive approach. A coding schema was developed based on the first three interviews, and refined iteratively. A subset of three interviews was analysed by all members of the research team independently to discuss and reach consensus for the coding framework. Codes were then grouped according to themes and subthemes. Reporting followed the Standards for Reporting Qualitative Research (SRQR) guidelines.^26^

## Results

Twenty-five interviews were undertaken with 21 doctors and four PNs, with a range of personal and professional characteristics, and from a variety of clinics, reflecting the breadth of primary care in Australia (Table 1).

**Table 1.** Participant and practice characteristics, *n* = 25

**Participant characteristics**
**Age, mean years (range)**	45 (31–67)
**Sex**	
Male	9 (36%)
Female	16 (64%)
**Country of primary medical or nursing degree**	
Australia	22 (88%)
UK	1 (4%)
Ireland	1 (4%)
New Zealand	1 (4%)
**Years working in general practice, mean (range)^a^**	14 (1–39)
**Full or part time**	
Full time	13 (52%)
Part time	12 (48%)
**Training level**	
GP fellow	16 (64%)
GP registrar	5 (20%)
PN	4 (16%)
**Proportion of usual patient load with children, mean % (range)**	24% (2–100%)
**Special interest in paediatrics**YesNo	11 (44%)
	14 (56%)
**Additional postgraduate qualification in paediatrics or child health**	
Diploma	5 (20%)
None	20 (80%)
**Practice characteristics**	
**Location**	
Inner urban	8 (32%)
Outer urban	3 (12%)
Regional	8 (32%)
Rural	4 (16%)
Remote	2 (8%)
**Number of full-time equivalent GPs at clinic, mean (range)**	6 (2–10)
**Number of full-time equivalent PNs at clinic, mean (range)**	2 (0–4)
**Type of clinic**	
Private	14 (56%)
Mixed community and private	6 (24%)
Community: Aboriginal health	3 (12%)
Community: Refugee health	1 (4%)
Corporate	1 (4%)

a Does not include hospital training. PN = practice nurse.

### Preferred collection method

Clean catch was the preferred collection method for a febrile child aged 6 months for just over half of the clinicians (*n* = 14), who mostly preferred it for its lower contamination than other non-invasive methods.

Urine bags were preferred by almost all of the remaining clinicians (*n* = 10), including all four PNs. Urine bags were mostly preferred for their convenience because clean catch was perceived to be time-consuming and difficult. One clinician reported preferring cotton wool balls owing to convenience.

No clinicians used invasive collection methods as first line. Invasive methods were regarded as impractical or unnecessary in primary care, and more suited to unwell children in the hospital setting. Some rural and remote clinicians reported using clean catch for most children, and catheter or SPA if the child was more unwell.

### Themes

**Figure 1.**
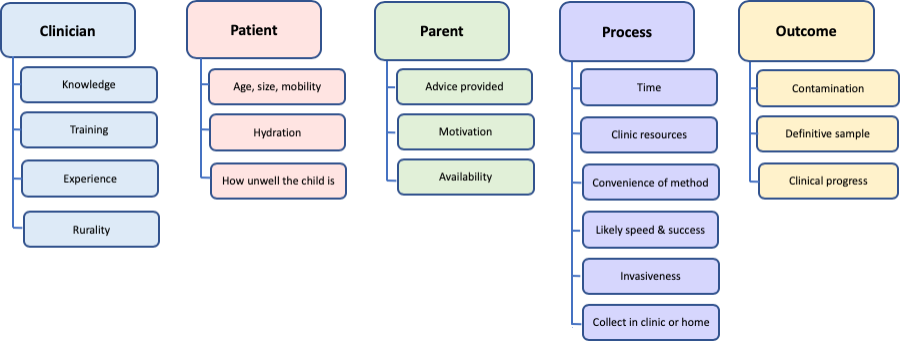
Barriers and enablers to urine sample collection from young children: themes and subthemes

Five main themes and 19 subthemes emerged from the analysis for the barriers and enablers to collecting urine samples from young children. These comprised: clinician’s knowledge and expertise; patient characteristics; parent or carer’s understanding and motivation; the collection process itself; and likely outcome of the chosen method (Figure 1). A list of further example quotes from each theme and subtheme are included in Supplementary Table 1.

### Clinician factors

#### Knowledge

Theoretical knowledge about collection methods was varied among clinicians, but knowledge of likely contamination rates, particularly for urine bags, was commonly reported:

*'And you can use urine bags, but, my understanding anyway, is that they’re much more prone to contamination. So we try to steer away from those if possible.'* (3, urban GP)

A minority of clinicians held differing views regarding likely contamination for bag samples:

*​'I find that I get very clean samples. And so yeah, I generally don't get any squames or very rarely in my samples, so I'm very happy with the accuracy of it.'* (4, urban GP)

#### Training

For many clinicians, their knowledge and preference for collection methods were strongly influenced by their previous training, both in hospital and in GP settings:

*​'Because of my training at the children's hospital when I was a young GP.'* (1, urban GP)

*'I think my GP supervisor when I was training, he did it. And that's where I learnt it.'* (14, urban GP)

#### Experience

Several clinicians learnt how to perform catheterisation or SPA during hospital paediatric training terms. However, infrequent ongoing practice in the GP setting resulted in them no longer feeling confident to perform these procedures:

*​ 'I haven’t done a suprapubic aspiration for a while now, so I could certainly try, I know the theory behind it, but I have a feeling that I may not be successful. So I think lack of practice, probably would slightly caution me.'* (17, urban GP)

#### Rurality

Geography and practice location frequently determined how likely GPs were to perform invasive collection methods. In urban and regional areas, many clinics were located close to a hospital:

*​'... being close to a paediatric emergency department I think I would be sending them in, rather than completing that procedure.'* (2, urban GP registrar)

In rural and remote areas, GPs often performed invasive collection procedures, as the clinicians working at the local hospital or in isolated locations:

*​'I think in general our rural colleagues do a lot more procedures than we do. Often they are on call for the base hospital.'* (17, urban GP)

*'I was in remote Queensland … So I did everything out there.'* (12, urban GP)

### Patient factors

#### Age, size, and mobility

Patient factors were a commonly mentioned barrier in relation to clean catch collection, particularly for older and more mobile children:

*'And certainly if the child is distressed and uncooperative and won't sit still, that's really tricky.'* (11, urban GP registrar)

#### Hydration

All clinicians considered hydration status to be important, with poor hydration being a barrier, and optimising hydration being an enabling strategy for collection success:

*'If they're really dehydrated, you're not going to get much urine out.'* (8, regional GP registrar)

#### How unwell the child is

The clinical state of the child strongly influenced whether clinicians felt the child should be referred to hospital for invasive sample collection and further assessment:

*​'If … something about their clinical examination makes me concerned, I don't think sending the family home would be appropriate in that setting, and I would send them off to the hospital.'* (17, urban GP)

### Parent or carer factors

#### Advice

The explanation and advice given to parents and carers were seen to strongly influence collection success, as in most cases parents enacted the actual collection process. Several clinicians felt it was important that parents understood why collecting a sample was important, as well as the logistics of the actual collection process:

*'... the better they understand that generally the more persistent they are, and successful getting the sample.'* (3, urban GP)

Printed handouts were identified as a valuable tool to educate and engage parents, enable collection success, and save time:

*'A lot of people take information in differently, and a lot of people are overwhelmed by words, and just words on a page, I think visual instructions are always helpful.'* (13, regional GP registrar)

#### Motivation

Many clinicians described the importance of parents being motivated to collect a sample, which was further enabled by parental health literacy. This was particularly the case for successful clean catch collection, which could be time-consuming or difficult:

*'So clearly some parents are really good at following instructions, you know, "Yes, we've gotta get this done," and others, I don't know, get caught up in other things.'* (11, urban GP)

#### Availability

However, for families with multiple children, or other resource limitations, clinicians recognised that collection could be challenging:

*​'... if they've got multiple other kids just the logistics of not being able to be two places at once.'* (7, regional GP registrar)

### Process factors

#### Time

Time availability in primary care was identified as a major barrier to sample collection, and influenced the choice of collection method:

*'Probably using the bag, simply because, time is often ... a bit pressed for time yeah. It's often very busy.'* (10, regional PN)

Several GPs discussed that if collection was going to take a long time, then in practice this would then need to happen at home:

*'… often it's a time-consuming thing trying to get a sample from an infant and it's time the doctors don't have.'* (24, rural PN)

#### Clinic resources

The availability of a suitable space also limited capacity for clean catch collection, and for invasive procedures:

*'You can't just sit them in the waiting room without their nappy on. I mean, you could but we generally choose not to because I think it would put other people off.'* (11, urban GP)

Separate treatment rooms or an unoccupied consulting room were an enabler, but this was available in a minority of clinics:

*​'GP clinics are run to make sure that rooms are occupied. So, room availability can be an issue.'* (13, regional GP registrar)

Some clinics had PNs who were able to help with sample collection:

*'In my practice its good because I have skilled nurses who can follow that up, so I can still be seeing other patients while we wait for that sample.'* (2, urban GP registrar)

However, often the nursing staff were also busy, or not available at the time required:

*​'… we're not very well staffed with nurses, and the nurse is super busy doing other things.'* (18, urban GP)

#### Convenience of method

Collecting urine samples from young children was generally regarded as a challenging process:

*'… as soon as you hear “fever” there’s always that little part of you that says “I bloody hope I can find a source here, because if I can’t find a source, it’s going to be a pain in the ass trying to get that urine sample.”'* (3, urban GP)

The perceived convenience of urine bags, for both the clinician and family, was a strong consideration for the clinicians who favoured their use:

*'I actually do like that bag collecting method. I think that's something that's a little easy. You can pop that on a child and perhaps set and forget for a bit, rather than standing there with a cup, hoping and waiting.'* (13, urban GP registrar)

In contrast clean catch was often seen to be inconvenient, often related to the attention required from parents:

*'In my experience, parents often say, “Oh, we can't do it,” or “It's too hard.”'* (1, urban GP)

#### Likely speed and success

Whether collection occurred in the clinic or at home, the likely speed and success of the collection method was a strong consideration for many clinicians. There was a consistent sense that clean catch collection was often time-consuming and, therefore, would often be something parents needed to do at home:

*'I think it's an excellent method, but it's obviously very time consuming.'* (4, urban GP)

While some clinicians felt that successful clean catch collection was very unlikely, others felt that most clean catch attempts were successful:

*​'... a clean catch is nearly impossible ... fluky if you can do it.'* (6, regional GP)

*'I've found most parents have come back with something.'* (7, regional GP registrar)

Some GPs reported difficulties with urine bag collection as well:

*'It is just difficult to get those urine bags to work, difficult to apply, difficult to get a clean sample. So I'm not sure what the percentage would be, but lots of the time the parent comes back and says it's just leaked and they haven't been able to get anything.'* (5, regional GP)

Clinicians were interested in ways of improving the speed and success of collection:

*'Definitely. If there were sort of practical, simple things that would make it easier to collect. Yes, I would be very keen to try that.'* (19, remote GP)

Voiding stimulation methods, such as the Quick-Wee technique,^23^ were felt to improve the speed and success of clean catch collection:

*'… one way to try and get the urine sample without having to wait for too long is to get the nappy off and then get a cool cloth and then rub in a circular motion just above the pubic bone where the bladder is, and sometimes it will induce a reflex where it starts urination, and you collect a sample from there.'* (3, urban GP)

Clinicians perceived these methods, when they knew about them, as simple to incorporate into practice, and acceptable to parents:

*'I learnt that in my hospital time, where I was doing it. And I think also from the nursing staff. Can't remember, but anyways, it's been quite successful.'* (21, urban GP registrar)

This timeliness of collection also influenced whether the child would need to attend hospital:

*'If we weren't getting a quick sample, I may consider referring them into the emergency department.'* (2, urban GP registrar)

#### Invasiveness

Using a catheter or needle to extract urine directly from the bladder was seen as highly invasive:

*'… there's the impression that it's, well, it's not a pleasant thing to do, it's really invasive, it's a bit of a nasty thing to do.'* (5, regional GP)

It was also thought that parents would not consider invasive methods acceptable outside the hospital setting, unless in rural or remote locations:

*​'I think the idea of a percutaneous intervention for an infant or baby is quite intimidating for a parent. They may not understand the relevance or appreciate the significance of it in a general practice setting, but in the hospital setting where there is generally a higher acuity of pathology, I think parents are more accepting of the need to undergo such a procedure.'* (17, urban GP)

#### Collection in clinic or at home

For the preferred non-invasive collection methods, clinicians varied in whether collection usually occurred in the clinic or at home:

*'I do try to get them to do that while they're in the clinic, which, again, most parents are pretty happy to do.'* (19, remote GP)

*'… well in our particular practice, um, we'll encourage them to catch the urine at home.'* (12, urban GP)

Collection at home also raised possible logistic challenges and concerns that parents may not return with the requested sample:

*'If you see them for example at 12:30 on a Saturday and the clinic centre closes at 2, how are you gonna deal with that if they manage to collect the specimen at 5 or 6, who's actually gonna do the dipstick and how would you deal with that.'* (1, urban GP)

Many clinicians reported initially trying to collect a sample in the clinic, but sending the child home for continued collection attempts if required:

*'We'll just get the jar ready and if they start to pee, we'll catch the specimen or then I'll give the jar to the parent and get them to wait and watch while I type up notes or do other things. And then if everything's done and we still don't get a specimen, then I might send them home to do it.'* (11, urban GP)

### Outcome factors

#### Contamination

Contamination was a recurring theme during interviews. Most clinicians were aware that urine bags had high contamination, but this did not always deter them from using them:

*'Well, probably what we would do is put one of the urine bags on, which I know is usually contaminated because of various things, but that's what we would do.'* (9, regional PN)

However, clinicians who preferred the clean catch method mostly did so because of its lower rate of contamination:

*'I would also explain to them the reason why the clean catch is important is the dilemma of if you get a contaminated specimen you're not sure what you're treating.'* (1, urban GP)

Some GPs felt that efforts should be made to discourage the use of urine bags, particularly for culture:

*​'Why are they still making bags for bag urine? If it's not accepted practice … remove the bag altogether because it just gives people the wrong idea.'* (17, urban GP)

#### Definitive sample

Collecting a sample that was ultimately likely to be definitive was often considered important. Many clinicians who preferred the clean catch method recognised that urine bags might be helpful for dipstick screening, but felt that urine bag culture would be unreliable owing to contamination and false-positives:

*'I groan because I think it’s only useful when it’s negative.'* (25, rural GP)

Where sample collection was difficult, some GPs might treat empirically for suspected UTI without a confirmatory sample:

*'… it’s not uncommon for GPs to treat empirically.'* (13, regional GP registrar)

#### Clinical progress

Often with time, further clinical information emerged influencing the ongoing need for a sample. Sometimes an alternate clinical focus developed, and some children simply got better with time:

*'If the child improves or things like that, and they haven't been able to get it, then I won't see them again. But then obviously the child has improved, and that’s OK.'* (4, urban GP)

Broadly, the barriers and enablers identified by clinicians in this study suggest four approaches to urine sample collection in primary care (Table 2). An evidence-based approach utilises clean catch for its lower contamination rate. A convenience approach favours urine bags given the time and space limitations of primary care. A pragmatic approach uses clean catch, but also uses urine bags in some circumstances. In rural and remote settings clean catch is used, but catheterisation or SPA are also performed when required for more unwell infants who cannot easily access hospital services.

**Table 2.** Approaches to urine sample collection in primary care

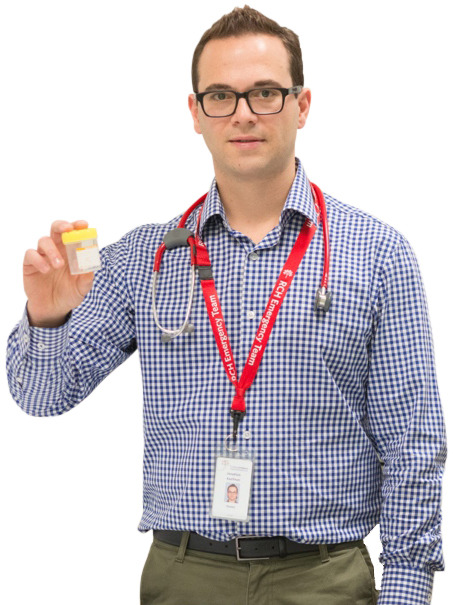	**Evidence based**Use clean catch methodAcknowledge can be time-consumingWant to avoid sample contamination
	**Convenience**Use urine bag methodConvenience for parent and clinicianAware of sample contamination
	**Pragmatic**Prefer clean catchUse urine bags sometimesDepends on clinical circumstances
	**Rural or remote**Prefer clean catchNot close to hospital servicesPerform catheterisation or SPA whenrequired

SPA = suprapubic aspiration.

## Discussion

### Summary

This study explored the barriers and enablers to collecting urine samples from young children in the primary care setting. While intending to identify barriers and enablers to collecting urine samples, what also emerged were how these factors influence the choice of collection method. Clinicians felt in general that collection was difficult and time-consuming, and non-invasive methods were universally preferred.

Barriers to collection included time and space constraints in clinics. Collection attempts were, therefore, often performed in the home environment by parents. Several enablers for successful sample collection were identified. These included patient hydration, parental motivation and parent education handouts, and the use of voiding stimulation techniques. Invasive methods were rarely performed except in rural and remote areas, and even GPs trained in these procedures felt they were unlikely to be acceptable to parents in an urban primary care setting.

Collection attempts were not always successful. For many children who had improved or for whom an alternate clinical focus had emerged, this was not detrimental, but for others this led to repeated clinical reviews, untargeted empiric treatment without sample confirmation, or referral to hospital for sample collection.

### Strengths and limitations

The study interviewed GPs and PNs from a diverse range of primary care clinical settings. Clinicians readily engaged with the study topic. Findings provide important insights into how clinicians choose and actually perform urine sample collection within the time, space, and resource constraints of primary care. While collection was often performed by parents, this study did not directly interview parents and carers to establish their views, which could also be valuable to inform the relevance and practicality of guideline recommendations.

The lead researcher being a paediatrician was a potential source of bias for the study. As with all qualitative studies, data analysis is subjective and can be influenced by the ideas and assumptions of the researcher. That the research team, which included a GP and a non-clinical researcher, reached consensus on coding and themes arising from the study suggests this was not a limitation to the study. Further, four clinicians were aware that the interviewer had developed the Quick-Wee voiding stimulation technique, but they did not necessarily use the method. Reflexive field notes did not suggest any personal bias influencing the interpretation of results.

### Comparison with existing literature

Results are consistent with previous surveys reporting that parents find non-invasive collection time-consuming and difficult,^18,19^ and that clinician preferences for collection methods vary.^10,27^

Urine bags are used widely in many countries.^10^ Although Australian guidelines recommend against culture of bag samples owing to the likelihood of contamination,^7^ in this study knowledge about contamination did not deter clinicians from using them.

Clinicians who used voiding stimulation methods found them easy to integrate into practice and acceptable to parents, consistent with previous studies in the emergency department setting.^23,24^ Several recent guidelines recommend voiding stimulation techniques, such as the Quick-Wee method, to improve the speed and success of clean catch collection.^7,28–30^

In the Australian and UK healthcare systems, primary care for children is mostly provided by GPs. In other countries such as the US, it is often provided by paediatricians. This may influence whether invasive collection methods are considered appropriate in the local primary care setting, or recommended in guidelines.^9^

### Implications for practice and research

The time, space, and resource constraints in primary care strongly influence how clinicians collect urine samples from young children. Non-invasive collection methods are favoured, but current non-invasive methods trade convenience for contamination. All collection methods have advantages and limitations, and there is no single approach suited to every patient and clinical situation. Clinicians must consider factors, such as the likelihood of collection success and the likelihood of collecting an uncontaminated specimen, when choosing the method of sample collection.

The ideal method would be fast, gentle, and simple, with low contamination. Further research is needed to optimise the speed, success, and low contamination of non-invasive collection. Where possible, clinicians choosing non-invasive collection should consider voiding stimulation and clean catch, to avoid the higher likelihood of contamination from pad and bag samples.

Some international guidelines recommend the use of invasive collection methods, which may be impractical in primary care in countries like Australia and the UK. Guidelines must consider the primary care context to ensure recommendations are relevant to real-world practice.
